# Shared Genetic Etiology between Alzheimer’s Disease and Blood Levels of Specific Cytokines and Growth Factors

**DOI:** 10.3390/genes12060865

**Published:** 2021-06-05

**Authors:** Robert J. van der Linden, Ward De Witte, Geert Poelmans

**Affiliations:** Department of Human Genetics, Radboud University Medical Center, P.O. Box 9101, 6500 HB Nijmegen, The Netherlands; Robert.vanderLinden@radboudumc.nl (R.J.v.d.L.); Ward.deWitte@radboudumc.nl (W.D.W.)

**Keywords:** Alzheimer’s disease, cytokines and growth factors, genome-wide association study (GWAS), Polygenic Risk Score (PRS)-based analysis

## Abstract

Late-onset Alzheimer’s disease (AD) has a significant genetic and immunological component, but the molecular mechanisms through which genetic and immunity-related risk factors and their interplay contribute to AD pathogenesis are unclear. Therefore, we screened for genetic sharing between AD and the blood levels of a set of cytokines and growth factors to elucidate how the polygenic architecture of AD affects immune marker profiles. For this, we retrieved summary statistics from Finnish genome-wide association studies of AD and 41 immune marker blood levels and assessed for shared genetic etiology, using a polygenic risk score-based approach. For the blood levels of 15 cytokines and growth factors, we identified genetic sharing with AD. We also found positive and negative genetic concordances—implying that genetic risk factors for AD are associated with higher and lower blood levels—for several immune markers and were able to relate some of these results to the literature. Our results imply that genetic risk factors for AD also affect specific immune marker levels, which may be leveraged to develop novel treatment strategies for AD.

## 1. Introduction

Alzheimer’s disease (AD) is a neurodegenerative disorder that causes patients to suffer from behavioral changes, a progressive decline in memory and cognitive function due to brain atrophy resulting from neuronal loss of function and death [1]. While AD is accountable for most dementia cases and affects millions of people worldwide, no disease-modifying therapies are currently available [2]. The neuropathological hallmarks of AD are extracellular plaques of amyloid-β (Aβ) and intracellular neurofibrillary tangles (NFT) composed of excessively phosphorylated tau [3]. The heritability of AD is estimated at 60–80% [4]. Dominant mutations in *APP*, *PSEN1* and *PSEN2* cause rare familial forms of AD characterized by an early onset (early-onset AD (EOAD) < 65 years) [4]. However, in the vast majority of cases, AD symptoms only manifest later in life (late-onset AD (LOAD) ≥ 65 years) and multiple genetic risk factors with small effect sizes contribute to LOAD development [4]. The strongest common genetic risk factor for LOAD is the ε4 allele of the apolipoprotein E (APOE) gene (*APOEε4*) [4]. Furthermore, environmental risk factors contribute to the multifactorial nature of AD. The mechanisms through which these risk factors affect biological pathways that ultimately result in AD are largely unknown. Elucidating the polygenic architecture of AD may therefore provide insights for the development of disease-modifying therapies.

Many of the LOAD candidate genes that were identified through genome-wide association studies (GWASs) are thought to play a role in regulating immunity, through their effect on microglial function [5,6,7]. Microglial cells are the resident macrophages of the brain and in addition to Aβ plaques and tau tangles, increased microglial activity and associated neuroinflammation has emerged as a third core pathology in AD [8]. Under physiological conditions, microglial cells survey the brain but only become activated and cause inflammation upon recognizing threats, such as infection, toxins and injury [8]. Although acute neuroinflammation could still serve as a defense mechanism against these threats, chronic neuroinflammation by excessively active microglial cells and recruitment of peripheral macrophages is detrimental to neuronal function [8]. The effects of peripheral macrophages may be exacerbated by impaired function of the blood–brain barrier (BBB) that separates the central nervous system from the rest of the body, and breakdown of the BBB is often observed in AD [9]. Moreover, activated microglial cells contribute to these detrimental effects by massively releasing inflammatory molecules and excessively pruning synapses, leading to synaptic loss [10]. In this respect, AD patient brains contain higher levels of activated, pro-inflammatory microglial cells [11,12]. In addition, the sustained activation of microglia and other immune cells has been demonstrated to exacerbate both Aβ and tau pathology [5,8]. Furthermore, genetic pleiotropy analyses have identified genetic overlap between AD and immune-mediated diseases—i.e., Crohn’s disease, psoriasis, and type 1 diabetes—indicating that aberrant immune processes influence AD pathogenesis and progression [12]. Chronic neuroinflammation in AD can be caused by both overexpression of pro-inflammatory cytokines and downregulation of anti-inflammatory cytokines that neutralize the harmful effects of chronic exposure to pro-inflammatory cytokines (reviewed in [13]). Moreover, the dysregulated immune system in AD is not limited to the central nervous system, and there is ample evidence for systemic immune signals (originating from outside the brain) contributing to AD (reviewed in [14]). Although all these data suggest a relationship between inflammation and AD, a (much) better understanding of this relationship could have implications for treatment and prevention strategies.

In this study, we investigated whether there is overlap between genetic risk factors—single nucleotide polymorphisms (SNPs)—for LOAD and SNPs contributing to the blood levels of a set of immune markers (cytokines and growth factors, inflammatory regulators that can be used as important intermediate phenotypes for inflammatory diseases [15]). To this end, we deployed shared genetic etiology and SNP effect concordance analyses, using publicly available GWAS results.

## 2. Materials and Methods

### 2.1. GWAS Summary Statistics for PRS-Based Analyses

For the polygenic risk score (PRS)-based analyses (see below), we used GWAS summary statistics from a Finnish AD cohort (1798 cases (diagnosed with ICD-10 code G301), 72,206 healthy controls) obtained through FinnGen (finngen_r4_AD_LO_EXMORE) as the ‘base’ sample. For the ‘target’ samples, we retrieved GWAS summary statistics for the blood levels of 41 immune markers (cytokines and growth factors) that were measured as a continuous phenotype in the general population from the study by Ahola-Olli et al. (GWAS sizes ranging from 840 to 8293 subjects; Table 1) [15].

### 2.2. PRS-Based Analyses

We performed PRS-based analyses with PRSice (v1.25), using the abovementioned Finish GWAS summary statistics as ‘base’ and ‘target’ samples [16] to determine the level of shared genetic etiology between AD and the blood levels of the 41 immune markers. First, we performed clumping based on the *p*-values of SNPs in the ‘base sample’ to select the most significant SNP among correlated SNPs that were in linkage disequilibrium (LD, R^2^ > 0.25) within a window of 500 kb using PLINK (v1.90) [17,18]. With PRSice we then calculated summary-level PRS by regressing the weights of selected AD risk SNPs (based on their *p*-value in the AD GWAS) on to the calculated weighted multi-SNP risk scores of immune marker blood levels, using the gtx package implemented in PRSice [16]. The PRS-based analyses were performed for all SNPs that exceeded seven default *p*-value thresholds (*P*_T_): 0.001, 0.05, 0.1, 0.2, 0.3, 0.4 and 0.5. A correction for multiple testing was then performed using a Bonferroni significance threshold < 0.05 (i.e., *p* < 0.05/287 tests (=7 *P*_T_s × 41 phenotypes) = 1.74 × 10^−4^).

### 2.3. SNP Effect Concordance Analyses

Subsequently, for those immune markers that we found to have a shared genetic etiology with AD, we performed SNP effect concordance analysis (SECA) to determine the direction of the genetic overlap [19]. We used SECA to calculate empirical *p*-values for the concordance between AD and immune marker blood levels, i.e., the agreement in the direction of the SNP effect across two phenotypes. We then performed a Bonferroni correction to account for the number of tests that we performed with SECA.

## 3. Results

### 3.1. PRS-Based Analyses

After correcting for multiple testing, we identified genetic sharing between AD and the blood levels of 15 out of the 41 immune markers (36.6%) for at least one of the seven used *p*-value thresholds (*P*_T_s) (Table 2). A complete overview of the PRS-based analyses including all *P*_T_s for all blood immune markers is provided in the Supplementary Materials (Table S1). For the 15 significant immune markers, genetic variants associated with AD also explained between 0.5 and 1.8% of the variation in their blood levels (Table 2).

### 3.2. SECA Analyses

SECA analyses showed a significant genetic concordance between AD and all 15 immune markers that emerged from our screening (Table S2). For the blood levels of seven of the 15 immune markers, we identified a positive concordance with AD, indicating that genetic risk factors associated with AD also contribute to increased blood levels of these markers (Table 2). For the other eight immune markers, we found a negative concordance with AD, implying that genetic risk factors for AD are also associated with lower blood levels of these markers (Table 2).

## 4. Discussion

In this paper, we identified genetic sharing between AD and the blood levels of 15 immune markers. Through concordance analyses, we also determined that for eight and seven of these markers, AD genetic risk factors contribute to increased and decreased blood levels, respectively.

First, we will discuss the literature findings about those immune markers for which we found a positive concordance between AD and their blood levels. Although no direct links between the blood levels of basic fibroblast growth factor (bFGF; other name: FGF2) have been reported, increased FGF2 levels were found in the brains of AD patients. In these brains, FGF2 was found within the neuritic plaques and in association with the neurofibrillary tangles that are characteristic of AD [20]. Further, FGF2 gene transfer restores hippocampal functions in mouse models of AD and viral delivery of FGF2 in the brain has been proposed as a therapeutic intervention for AD [21], further indicating an important role for FGF2 in AD. Granulocyte colony-stimulating factor (GCSF) stimulates the production and release of neutrophils in the blood and is also a neurotrophic factor [22]. In this respect, it is interesting that decreased GCSF blood levels have been reported in AD patients, although among these patients, higher GSCF blood levels associate with increased disease severity [22]. However, while this latter finding is in keeping with the positive genetic concordance between AD and blood GCSF levels that we observed, GCSF treatment has also been shown to improve memory in an AD rat model [23]. In addition, again in line with our findings, higher levels of hepatocyte growth factor (HGF)—which regulates various brain functions, including axonal outgrowth, neuronal survival, and synaptic plasticity—have been found in the blood, cerebrospinal fluid (CSF) and brains of AD patients [24,25]. In this respect, increased HGF immunoreactivity within neurons, astrocytes and microglial cells was also demonstrated to be an indicator of gliosis and microglial proliferation that occurs around Aβ plaques in AD brains [25]. In contrast to the results of our concordance analysis, decreased blood levels of stem cell factor (SCF) have been described in AD patients, and these decreased levels are also associated with a higher rate of cognitive decline [26]. 

In addition to the four abovementioned growth factors, we identified a positive genetic concordance between AD and the blood levels of three cytokines: interleukin 10 (IL10), IL12p70 and CCL4. As for IL10, this cytokine is a negative regulator of the innate immune system and *IL10* knockout in an AD mouse model resulted in increased Aβ clearance by activated microglia and a partially rescued synaptic integrity in the brains of these mice [27]. The negative role of IL10 in AD is corroborated by our finding that genetic variants associated with AD also contribute to increased IL10 blood levels. Furthermore, in the brains of AD patients, significantly increased levels of both the anti-inflammatory IL10 and the pro-inflammatory IL12p70 have been reported, indicating that both anti- and pro-inflammatory signaling can be activated simultaneously in AD [28]. In addition, IL12p70 has been shown to reduce neuronal viability in cell culture experiments, both in the presence or absence of Aβ [29]. Lastly, chronic inflammation leads to elevated CCL4 levels in AD brains [30], while CCL4 levels are also increased in microglia associated with Aβ plaques [31].

For eight immune markers (three growth factors and five cytokines), we found a negative concordance between AD and their blood levels. Firstly, nerve growth factor (NGF) contributes to the survival, regeneration, and death of neurons during aging and in neurodegenerative diseases such as AD [32]. Impaired NGF signaling has also been linked to neurons losing their cholinergic phenotype in the AD basal forebrain [33] and brain implants delivering NGF to the cholinergic basal forebrain are currently being tested as an AD treatment in humans [34]. In this respect, it is interesting that we found that genetic variants associated with AD contribute to decreased NGF levels in the blood, which may reflect what is happening with NGF levels in the brain. Further, macrophage migration inhibitory factor (MIF) levels have been found to be increased in the blood, CSF and brains of AD patients [35]. Although the finding about MIF levels in the blood of AD patients is opposite to the negative genetic concordance that we identified, MIF colocalizes with Aβ plaques and increased MIF levels protect neuronal cells from Aβ-induced neurotoxicity [36]. Hence, MIF levels may be upregulated in the brain as a defense mechanism to compensate for declined cognitive function in AD [36]. Moreover, although no direct links between blood SCGFβ levels and AD have been reported, it is interesting that blood IL10 levels—for which we found a positive concordance with AD—negatively predict blood SCGFβ levels [37], which is in line with the negative concordance with AD that we observed.

As for the cytokines for which we found a negative concordance between AD and their blood levels, microvessels from AD brains produce and release high levels of CCL3 compared to control brains, suggesting that the brain microvasculature contributes to the inflammatory environment of the AD brain through upregulating CCL3 expression [38]. In addition, elevated levels of CCL5 in the cerebral microcirculation of AD patients were reported, and CCL5 treatment of neurons increases cell survival, suggesting a neuroprotective role for CCL5 [39,40]. Moreover, monocytes from AD patients produce significantly higher amounts of CXCL1 compared to age-matched controls, which causes these monocytes to migrate from the blood to the brain [41]. In the brain, CXCL1 also promotes the cleavage of tau, which is considered an early event in AD development [42]. Taken together, the literature findings on the relationship between AD and the brain levels of CCL3, CCL5 and CXCL1 are opposed to the negative concordance between AD and their blood levels that we found. In this respect, there may be an inverse relationship between the blood and brain levels of these cytokines, which we could speculate would, e.g., result from the recruitment of cytokine-producing immune cells to the site of inflammation—i.e., from the blood to the brain in the case of AD—that would in turn lead to a relative depletion of the cytokines in the blood. Incidentally, this may also apply to the other immune markers for which we found a discrepancy between the literature findings and the results from our concordance analysis—such as SCF, see above—and in fact, a recent study found that there were relatively few direct correlations between blood and CSF levels of cytokines in multiple neuro-inflammatory diseases [43]. Further, TRAIL is specifically expressed in the brains of AD patients and completely absent in the brains of healthy controls [44], and anti-TRAIL antibodies reduce brain Aβ load and improve cognition in an AD mouse model [45]. However, it was also reported that TRAIL blood levels do not differ between AD patients and controls [46]. Lastly, blood IL8 levels were found to be decreased in AD patients—consistent with the negative genetic concordance that we observed—while both lower and higher IL8 levels have been reported in the CSF of AD patients [47,48]. In addition, IL8 promotes inflammation and cell death of cultured neurons [49] while, in contrast, neurons also produce IL8 as a protection against Aβ-induced toxicity [50].

This study has two main limitations. First, genetic sharing between AD and blood levels of immune markers does not necessarily mean higher or lower immune marker blood levels are causative of AD. Second, as already indicated, blood levels may not reflect what is happening in the brain directly and there may also be an inverse relationship between the levels of immune markers in the blood and CSF, which warrants further investigation using CSF and post-mortem brain samples. This being said, we can conclude that genetic risk factors for AD also affect the blood levels of specific immune markers, suggesting that systemic immune processes may influence AD pathogenesis and progression. Although further studies are needed to confirm our findings, and depending on whether AD shows genetic overlap with increased or decreased immune marker blood levels, novel treatment strategies for AD could be developed.

## Figures and Tables

**Table 1:** GWAS summary statistics for the blood levels of 41 immune markers in the general population that were used in this study were obtained from the study by Ahola-Olli et al.

Name	Description	Type	N GWAS
CCL11	Eotaxin	Cytokine	8153
CCL2 (MCP1)	Monocyte chemotactic protein-1	Cytokine	8293
CCL27 (CTACK)	Cutaneous T-cell attracting	Cytokine	3631
CCL3 (MIP1α)	Macrophage inflammatory protein-1α	Cytokine	3522
CCL4 (MIP1β)	Macrophage inflammatory protein-1β	Cytokine	8243
CCL5 (RANTES)	Regulated upon activation, normal T cell expressed and secreted	Cytokine	3421
CCL7 (MCP3)	Monocyte specific chemokine 3	Cytokine	843
CXCL1 (GROa)	Growth-regulated oncogene-α	Cytokine	3505
CXCL10 (IP10)	Interferon γ-induced protein 10	Cytokine	3685
CXCL12 (SDF1a)	Stromal cell-derived factor-1 α	Cytokine	5998
CXCL9 (MIG)	Monokine induced by interferon-γ	Cytokine	3685
FGF2 (FGFBasic)	Basic fibroblast growth factor	Growth factor	7565
GCSF	Granulocyte colony-stimulating factor	Growth factor	7904
HGF	Hepatocyte growth factor	Growth factor	8292
IFNγ	Interferon-γ	Cytokine	7701
IL10	Interleukin-10	Cytokine	7681
IL12p70	Interleukin-12p70	Cytokine	8270
IL13	Interleukin-13	Cytokine	3557
IL16	Interleukin-16	Cytokine	3483
IL17	Interleukin-17	Cytokine	7760
IL18	Interleukin-18	Cytokine	3636
IL1b	Interleukin-1-β	Cytokine	3309
IL1ra	Interleukin-1 receptor antagonist	Cytokine	3638
IL2	Interleukin-2	Cytokine	3475
IL2ra	Interleukin-2 receptor, α subunit	Cytokine	3677
IL4	Interleukin-4	Cytokine	8124
IL5	Interleukin-5	Cytokine	3364
IL6	Interleukin-6	Cytokine	8189
IL7	Interleukin-7	Cytokine	3409
IL8 (CXCL8)	Interleukin-8	Cytokine	3526
IL9	Interleukin-9	Cytokine	3634
MCSF	Macrophage colony-stimulating factor	Growth factor	840
MIF	Macrophage migration inhibitory factor (glycosylation-inhibiting factor)	Growth factor	3494
PDGFbb	Platelet-derived growth factor BB	Growth factor	8293
SCF	Stem cell factor	Growth factor	8290
SCGFβ	Stem cell growth factor β	Growth factor	3682
TNFα	Tumor necrosis factor-α	Growth factor	3454
TNFβ	Tumor necrosis factor-β	Growth factor	1559
TRAIL	TNF-related apoptosis-inducing ligand	Cytokine	8186
VEGF	Vascular endothelial growth factor	Growth factor	7118
βNGF	β nerve growth factor	Growth factor	3531

NOTE: Alternative names are indicated between brackets. Abbreviation: GWAS, genome-wide association study.

**Table 2:** Fifteen immune markers for which we identified genetic sharing between AD and their blood levels.

Immune Marker	Best *P*_T_	N SNPs	Bonferroni *p*-Value	Variance Explained R^2^	Concordance with AD
CCL4 (MIP1β)	0.001	2001	3.51 × 10^−5^	0.003227	+
FGF2 (FGFBasic)	0.5	497,296	7.18 × 10^−7^	0.004509	+
GCSF	0.2	236,031	3.45 × 10^−3^	0.002255	+
HGF	0.5	498,823	4.25 × 10^−4^	0.002631	+
IL10	0.5	497,485	1.38 × 10^−7^	0.004857	+
IL12p70	0.5	498,327	7.70 × 10^−3^	0.001971	+
SCF	0.5	498,841	3.14 × 10^−3^	0.002171	+
bNGF	0.001	1894	4.03 × 10^−3^	0.004956	-
CCL3 (MIP1α)	0.05	65,371	2.25 × 10^−2^	0.004048	-
CCL5 (RANTES)	0.3	310,864	2.45 × 10^−8^	0.011848	-
CXCL1 (GROα)	0.2	221,410	1.54 × 10^−13^	0.018177	-
IL8	0.2	220,931	9.84 × 10^−5^	0.006968	-
MIF	0.2	221,727	6.80 × 10^−5^	0.007234	-
SCGFβ	0.001	1927	1.54 × 10^−4^	0.006441	-
TRAIL	0.3	332,200	3.18 × 10^−5^	0.003273	-

Abbreviations: *P*_T_, *p*-value threshold; SNP, single nucleotide polymorphism.

## Data Availability

FinnGen Alzheimer’s disease GWAS summary statistics can be accessed online at: https://www.finngen.fi/en/access_results (accessed on 11 May 2021). Cytokine and growth factor GWAS summary statistics from the study by Ahola-Olli et al. can be accessed online at: https://www.ebi.ac.uk/gwas/publications/27989323 (accessed on 11 May 2021).

## Supplementary Materials

The following are available online at https://www.mdpi.com/article/10.3390/genes12060865/s1, Table S1: Shared genetic etiology analyses between AD and the blood levels of 41 immune markers across seven *p*-value inclusion thresholds (PTs) using a polygenic risk score (PRS)-based approach, Table S2: SNP effect concordance (SECA) analyses on the genetic concordance between AD and blood immune marker levels that were significant in the polygenic risk score (PRS)-based analyses.
